# Concentrations of tamoxifen and its major metabolites in hormone responsive and resistant breast tumours

**DOI:** 10.1054/bjoc.2000.1120

**Published:** 2000-04-27

**Authors:** J MacCallum, J Cummings, J M Dixon, W R Miller

**Affiliations:** Edinburgh Breast Unit Research Group ICRF Medical Oncology Unit Edinburgh Breast Unit, Western General Hospital, Edinburgh, EH4 2XU, UK

**Keywords:** tamoxifen, 4-hydroxytamoxifen, desmethyltamoxifen, response

## Abstract

Patients treated with tamoxifen (TAM) for primary breast cancer often manifest de novo or acquired resistance, possibly through changes in drug metabolism. Using solid-phase extraction methods and reversed-phase high-performance liquid chromatography separations, levels of TAM and metabolites 4-hydroxytamoxifen (4OH) and desmethyltamoxifen (DMT) have been measured in plasma and tumour tissue from breast cancer patients treated with TAM for at least 3 months. Patients were categorized into those with tumours responding to TAM and those showing de novo or acquired resistance. Levels of TAM, 4OH and DMT in both plasma and tissue samples were correlated with clinical response, length of treatment and patient weight. Interesting results included accumulation of 4OH in tumour tissues over time in all patients, with significance reached in the acquired resistance group. In addition, significantly lower levels of 4OH and DMT were found in plasma taken from responding patients after 3 months of treatment when compared to non-responding patients, and a small group of ER-poor patients showed significantly lower levels of all three species in plasma when compared to other patients. Whilst not explaining TAM resistance in all cases, these differences could account for the development of resistance to TAM treatment in certain subgroups of patients. © 2000 Cancer Research Campaign


British Journal of Cancer (2000) **82**(10), 1629–1635
© 2000 Cancer Research Campaign
DOI: 10.1054/ bjoc.2000.1120, available online at http://www.idealibrary.com on IDEAL®

### Concentrations of tamoxifen and its major metabolites in hormone responsive and resistant breast tumours

**J MacCallum[1], J Cummings[2], JM Dixon[3] and WR Miller[1]**

[1]Edinburgh Breast Unit Research Group, [2]ICRF Medical Oncology Unit and the [3]Edinburgh Breast Unit, Western General Hospital, Edinburgh EH4 2XU, UK

**Summary** Patients treated with tamoxifen (TAM) for primary breast cancer often manifest de novo or acquired resistance, possibly through changes in drug metabolism. Using solid-phase extraction methods and reversed-phase high-performance liquid chromatography separations, levels of TAM and metabolites 4-hydroxytamoxifen (4OH) and desmethyltamoxifen (DMT) have been measured in plasma and tumour tissue from breast cancer patients treated with TAM for at least 3 months. Patients were categorized into those with tumours responding to TAM and those showing de novo or acquired resistance. Levels of TAM, 4OH and DMT in both plasma and tissue samples were correlated with clinical response, length of treatment and patient weight. Interesting results included accumulation of 4OH in tumour tissues over time in all patients, with significance reached in the acquired resistance group. In addition, significantly lower levels of 4OH and DMT were found in plasma taken from responding patients after 3 months of treatment when compared to non-responding patients, and a small group of ER-poor patients showed significantly lower levels of all three species in plasma when compared to other patients. Whilst not explaining TAM resistance in all cases, these differences could account for the development of resistance to TAM treatment in certain subgroups of patients. © 2000 Cancer Research Campaign

**Keywords:** tamoxifen; 4-hydroxytamoxifen; desmethyltamoxifen; response

As a non-steroidal anti-oestrogen, tamoxifen (TAM) is frequently used as endocrine therapy for human breast cancer (Tormey et al, 1976; Robinson and Jordan, 1988; Lerner and Jordan, 1990), and whilst it is effective in many patients (approximately two-thirds of oestrogen receptor (ER)-rich patients), the majority of unselected cancers appear resistant, with those which do respond to therapy eventually relapsing (Raam et al, 1988; Gottardis et al, 1989; Graham et al, 1990; Scott et al, 1991; Osborne et al, 1991, 1992; Wolf et al, 1993; Horwitz, 1993).

Resistance to TAM therapy may be due to a single, or a combination of several factors including; down-regulation or loss of oestrogen receptors (Miller et al, 1984; Raam et al, 1988; Murphy and Dozlaw, 1989; Graham et al, 1990; Foster et al, 1991; Scott et al, 1991; Horwitz, 1993); altered production of growth factors such as transforming growth factor beta (TGF-β) (Miller et al, 1984; Koga and Sutherland, 1987); altered signal transduction and increased expression of anti-oestrogen binding sites (AEBSs) which may directly mediate anti-oestrogen actions (Murphy and Sutherland, 1981; Miller and Katzenellenbogen, 1983). Pharmacological mechanisms such as a shift in metabolism, producing isomers (Wolf et al, 1993) or oestrogenic metabolites, and changes in levels of drug accumulation within tumour tissues (Osborne et al, 1987, 1991, 1992) may additionally be important.

Although significant differences in levels of circulating TAM and metabolites have not been reported between responding and resistant tumours (Johnstone et al, 1993; MacCallum et al, 1996), TAM resistance and stimulation of growth may be associated with a reduction in intra-tumoural TAM levels (Gottardis and Jordan, 1988; Osborne et al, 1991, 1992), with stimulation of growth on increasing concentrations in a dose-dependent manner (Gottardis et al, 1989). In breast cancer patients, TAM and its metabolites accumulate in tumour tissue when compared to serum or plasma (Daniel et al, 1981; Lien et al, 1991*a*, 1991*b*), and intra-tumoural levels thus do not necessarily reflect those in the circulation.

Having developed a rapid, sensitive and selective solid-phase extraction (SPE) and a high-performance liquid chromatography (HPLC) separation method for the determination of TAM and its major metabolites in plasma (MacCallum et al, 1996) and tumour (MacCallum et al, 1997) tissue, the aim of the present study was to correlate levels in tumour tissue and plasma with response to TAM therapy in a group of breast cancer patients.

### MATERIALS AND METHODS

TAM base (z-type isomer), *cis*-TAM (CIS, *e*-type isomer) and metabolites 4OH and DMT were generously supplied by Zeneca Pharmaceuticals (Macclesfield, UK). [N-methyl-$^3$H]TAM was obtained from Amersham International (Little Chalfont, UK). Standard solutions were prepared in methanol (MeOH) and stored at 4°C. MeOH and acetonitrile (ACN) were of HPLC-reagent grade (Rathburn Chemicals, Walkerburn, UK); triethylamine (TEA) and sodium chloride (NaCl) were obtained from Sigma (Poole, UK) and dimethylsulphoxide (DMSO) was analytical grade (Fisons, Loughborough, UK). Water was deionized and bi-distilled in a milli-U10 water purification system (Millipore, Harrow, UK). Solid-phase extraction was carried out on Bond-elut $C_2$ 3-cm$^3$ columns (Varian sample preparation products, Phenomenex, Macclesfield, UK). The HPLC stationary phase was μbondapak $C_{18}$ (125Å, 10 μm) packed in a 30 cm × 3.9 mm I.D. stainless steel column with a 1 cm $C_{18}$ guard column (Waters, Watford, UK).

*Received 5 January 1999*
*Revised 11 November 1999*
*Accepted 13 February 2000*

*Correspondence to:* J MacCallum, Department of Life Sciences, Napier University, Merchiston Campus, Colinton Road, Edinburgh EH10 5DT, UK

### HPLC

The chromatography systems used are detailed in MacCallum et al (1996, 1997). For plasma measurement the mobile phase consisted of 1% TEA (in $dH_2O$, pH 8.0) in MeOH (11:89, v/v), and for tumour measurement of 1% TEA (in $dH_2O$, pH 9.0) in MeOH (11:89, v/v). Mobile phase components were passed through a 0.22 μm filter and degassed prior to use. Elution was isocratic at a flow-rate of 1.2 ml $min^{-1}$ at ambient room temperature.

### Patient data

Samples were obtained from patients treated in the Edinburgh Breast Unit. Plasma and primary tumour tissue was obtained at surgery from 54 post-menopausal, ER-rich (> 20 fmol $mg^{-1}$ cytosol protein) breast cancer patients treated with tamoxifen for at least 3 months (range 3–86 months). Response at 3 months and at surgery (if > 3 months) was monitored by monthly ultrasound and clinical measurement of the tumour. Patients were classified as responding where tumour volume decreased by at least 25% between initial biopsy and the chosen timepoint (3 months and surgery), whilst non-responders showed either no change (< 25% increase or < 25% decrease in volume) or an increase (> 25%) in tumour volume (Forouhi et al, 1994).

Patients were then categorized according to response of their tumour throughout treatment. Thus, tumours decreasing in size with continual reduction or decreasing then showing no change thereafter were considered to be responders (R-R, *n* = 25); those initially reducing in size but which later increased in size were considered to have developed acquired resistance to tamoxifen (R-NR, *n* = 22; minimum length of treatment 4 months); and tumours remaining the same size or increasing in size on treatment were considered to show de novo resistance (NR-NR, *n* = 6).

### Sample preparation

This was carried out as detailed in MacCallum et al (1996, 1997). Briefly, 1 ml aliquots of plasma, and 0.5 mg aliquots of tumour tissue (all tissue available was dismembrated and 0.5 mg aliquots resuspended in DMSO), were spiked with radioinert CIS (and $^3H$ TAM in the case of tumour tissue) in order to monitor extraction efficiency. Aliquots were then applied to an activated solid-phase extraction column, which was washed through with $dH_2O$, $dH_2O$:MeOH (50:50) and ACN. Elution was with 1 M NaCl: MeOH (5:95, v/v). Samples were then dried, resuspended and analysed by HPLC. For tumour tissue, an aliquot of the resuspended eluent was placed into scintillant to count $^3H$ TAM for an additional calculation of recovery.

Analyses were carried out in duplicate in the presence of blank plasma and tumour samples, spiked plasma and tumours samples and standards in MeOH as controls. Multiple analyses were carried out to determine reproducibility. Accuracy (± precision) was 111–168% (± 43–63%) for plasma measurement at 10 ng $ml^{-1}$, and 63–94% (± 13–26%) for tumour measurement at 20 ng $g^{-1}$. Plasma measurements showed a tendency for overestimation at lower concentrations, with greater accuracy at higher concentrations (98–109% ± 5–10% at 1 μg $ml^{-1}$), although there was still a tendency for overestimation of 40H levels.

Concentrations of plasma and tumour were calculated from the peak areas measured by HPLC (Figure 1), and normalized to 100% taking into account extraction efficiency and any day-to-day alteration (monitored via spiked samples), and were expressed as ng $ml^{-1}$ of plasma and ng $g^{-1}$ of tissue. Extraction efficiency (± between-day variation) was 59–64% (±2.4–14.5%) at 2 ng $ml^{-1}$ for plasma measurements and 60–64% (±16–20%) at 20 ng $g^{-1}$ for tumour measurements.

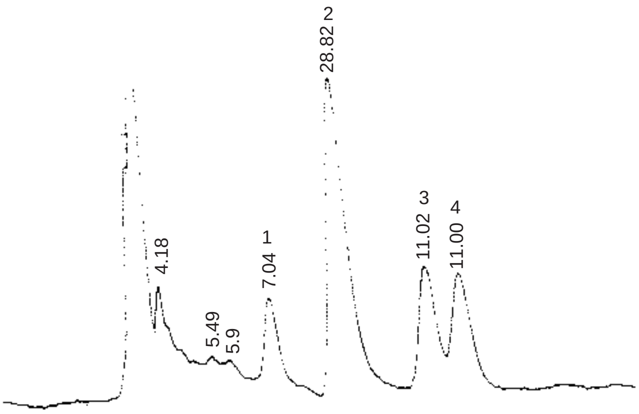

**Figure 1** An example of an HPLC trace, obtained from a tumour sample prepared by solid-phase extraction. Peaks relate to: 1 = 4OH; 2 = DMT; 3 = TAM; 4 = CIS (*cis*-isomer of tamoxifen). Drug levels are calculated from the measured area under each peak

### Statistical analysis

Levels of TAM, 4OH and DMT in blood and tumour tissue were calculated as described previously (MacCallum et al, 1996, 1997), and statistical analysis was carried out to determine associations between metabolite levels and: length of time on tamoxifen; response to tamoxifen at 3 months, final surgery and overall; and weight of patient at presentation (where available). In addition, tumour:plasma ratios were correlated with time and response; and the possible effects of tumour cellularity were examined by correlation with ER values.

Analyses on the mean concentrations of each metabolite were carried out using non-parametric tests, as the data were not normally distributed. Tests included Spearman rank, Mann–Whitney, Kruskal–Wallis and Pearson tests, correlation and regression analysis.

### RESULTS

Levels of TAM, 4OH or DMT in blood plasma and tumour tissue were correlated with treatment time in both a continuous manner, and additionally using arbitrary time divisions (< 5 months, 5–11 months and > 11 months of treatment) (Table 1). Although there was no significant accumulation of plasma or tumour TAM or DMT levels with time, levels of 4OH in tumour tissues showed a tendency to increase with increasing time on TAM (Kruskal–Wallis, $P$ = 0.066), whilst tumours from patients receiving < 5 months of treatment were found to contain significantly less 4OH when compared to those receiving > 11 months of treatment (Mann–Whitney, $P$ = 0.02). Additionally, in those patients showing overall acquired resistance to tamoxifen, there was significant accumulation of 4OH continuously over treatment

© 2000 Cancer Research Campaign

**Table 1** Correlations with length of treatment and initial patient weight

| Time on TAM (months) | | 4OH Tumour (ng g$^{-1}$) | 4OH Plasma (ng ml$^{-1}$) | DMT Tumour (ng g$^{-1}$) | DMT Plasma (ng ml$^{-1}$) | TAM Tumour (ng g$^{-1}$) | TAM Plasma (ng ml$^{-1}$) |
|---|---|---|---|---|---|---|---|
| All patients | (*n* = 54) | 156 (± 134) | 67 (± 34) | 569 (± 689) | 235 (± 116) | 331 (± 250) | 115 (± 63) |
| < 5 | (*n* = 15) | 92 (± 79) | 57 (± 22) | 612 (± 911) | 231 (± 97) | 331 (± 181) | 116 (± 43) |
| 5–11 | (*n* = 19) | 159 (± 129) | 79 (± 28) | 443 (± 484) | 239 (± 84) | 302 (± 220) | 115 (± 56) |
| > 11 | (*n* = 20) | 200 (± 156) | 60 (± 41) | 657 (± 681) | 241 (± 151) | 358 (± 322) | 124 (± 74) |
| Initial weight (kg) | | | | | | | |
| All patients | (*n* = 34) | 147 (± 120) | 65 (± 28) | 611 (± 831) | 235 (± 98) | 326 (± 247) | 115 (± 48) |
| < 63 | (*n* = 12) | 132 (± 118) | 68 (± 31) | 257 (± 232) | 247 (± 116) | 273 (± 122) | 139 (± 48) |
| 63–69 | (*n* = 8) | 129 (± 90) | 64 (± 15) | 624 (± 919) | 226 (± 92) | 345 (± 322) | 102 (± 44) |
| > 69 | (*n* = 13) | 174 (± 141) | 63 (± 34) | 857 (± 1086) | 245 (± 79) | 367 (± 293) | 99 (± 43) |

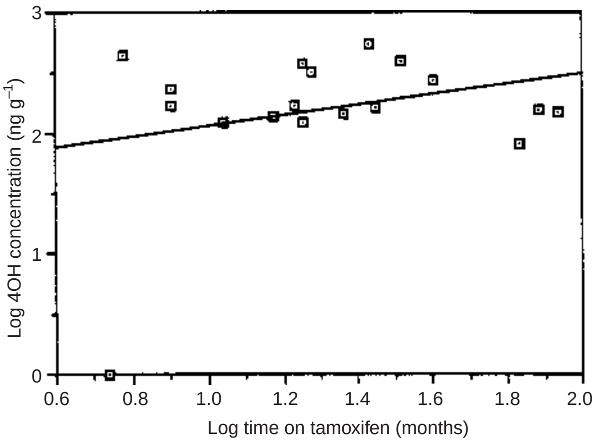

**Figure 2** Tissue levels of 4OH in tumours showing acquired resistance plotted against length of time of treatment (*n* = 22, *P* = 0.017)

duration (R-NR, Spearman rank, *P* = 0.017) (Figure 2). In contrast, plasma concentrations of 4OH were found to be significantly higher in the 5–11 months treatment group, when compared to the other arbitrary groupings (Mann–Whitney, *P* = 0.04, Table 1).

All patients were treated with the same dose of TAM (20 mg day$^{-1}$). However, there is evidence from an overlapping study on patients treated at the Edinburgh Breast Unit, that there is a correlation between TAM levels and weight, especially in the upper quartile (Love et al, 1997). Thus, where available (*n* = 32), we examined patient weight (kg) at time of presentation in association with TAM and metabolite levels. There were no significant correlations between weight and levels of 4OH or DMT although TAM levels did show a tendency to increase with increasing weight and time in tumours exhibiting acquired resistance (R-NR, regression analysis, *r* = 0.03). When patients were divided arbitrarily into three groups; < 63, 63–69 and > 69 kg initial weight, again no significant differences were apparent (Table 1). Further, when weight was taken into account, mean concentrations of TAM, 4OH and DMT in plasma (ng ml$^{-1}$ kg$^{-1}$) and tumour (ng g$^{-1}$ kg$^{-1}$) showed no significant differences between the three response groups.

Where levels of TAM, 4OH and DMT in tumour were correlated with response after 3 months of treatment, and at surgery, there were no significant differences between tumours which either responded or did not respond to TAM (Table 2). However, similarly grouped plasma levels showed differences between responding and non-responding patients after 3 months of TAM. Plasma levels of 4OH and DMT were significantly higher in non-responding patients (Mann–Whitney, *P* = 0.05, *P* = 0.03). No such differences were seen for TAM after 3 months of treatment, or for any of the three species in plasma taken at surgery.

Patients were additionally grouped according to overall tumour response (Table 3). Once again, no significant differences were seen in plasma or tumour levels of TAM, 4OH or DMT in relation to response type, the exception being significantly higher levels of DMT in the plasma of the de novo resistant group when compared to the responding group (Mann–Whitney, *P* = 0.03). Tumour:plasma ratios were calculated to evaluate tumour accumulation of TAM, but once again no significant differences were seen between response groups.

To examine the inter-relationships between metabolite levels, tumour concentrations of each metabolite were plotted against each of the others. On doing this we noted significant associations between TAM and 4OH in de novo resistant tumours, and between TAM and DMT in responding and acquired resistance tumours (Figure 4).

Levels of TAM and metabolites were wide-ranging throughout the group of patients, which may reflect the large heterogeneity of breast cancers, and the presence of areas of non-neoplastic cells within our tissue samples. Whilst we attempted to minimize this effect during our sample preparation, we were unable to correct entirely for differences in tissue cellularity. ER values (fmol mg$^{-1}$ cytosol protein), were known in 41 samples. No correlations were evident with metabolite levels (mg ml$^{-1}$ or mg g$^{-1}$) (data not shown). Interestingly, two samples taken from involved lymph nodes were available, in both of which concentrations of TAM, 4OH and DMT had accumulated to very high levels (Table 3).

Other samples examined included a number of mucoid cancers, in which cases, response to treatment is difficult to ascertain by clinical and ultrasound measurement. No significant differences in concentrations of TAM, 4OH and DMT were noted when comparing the three response groups (Table 3). Additionally a small number of samples were available from ER-poor patients who had received short-term TAM therapy (Table 3). Tumour metabolite levels did not differ significantly from the other response groups, however, whilst 4OH plasma levels did not differ greatly in the three main response groupings, levels in ER-poor patient plasmas were significantly lower than all other subgoupings (apart from mucoid tumours)

© 2000 Cancer Research Campaign

**Table 2** Mean levels of TAM, 4OH and DMT in responding (R) and non-responding (NR) patient plasma and tumour after 3 months of treatment, and at final surgery

| | 3 months treatment | | Final surgery | |
|---|---|---|---|---|
| **Tumour**[a] | **R ($n$ = 47)** | **NR ($n$ = 6)** | **R ($n$ = 25)** | **NR ($n$ = 28)** |
| 4OH | 157 (± 137) | 145 (± 118) | 138 (± 107) | 171 (± 154) |
| DMT | 589 (± 733) | 436 (± 215) | 610 (± 875) | 534 (± 489) |
| TAM | 340 (± 265) | 273 (± 100) | 334 (± 233) | 327 (± 268) |
| Plasma[b] | | | | |
| 4OH | 68 (± 35) | 74 (± 23) | 66 (± 30) | 80 (± 78) |
| DMT | 227 (± 116) | 313 (± 80) | 224 (± 98) | 242 (± 123) |
| TAM | 117 (± 63) | 131 (± 30) | 118 (± 58) | 115 (± 58) |

[a] ng $g^{-1}$ ± s.d. [b]ng $ml^{-1}$ ± s.d.

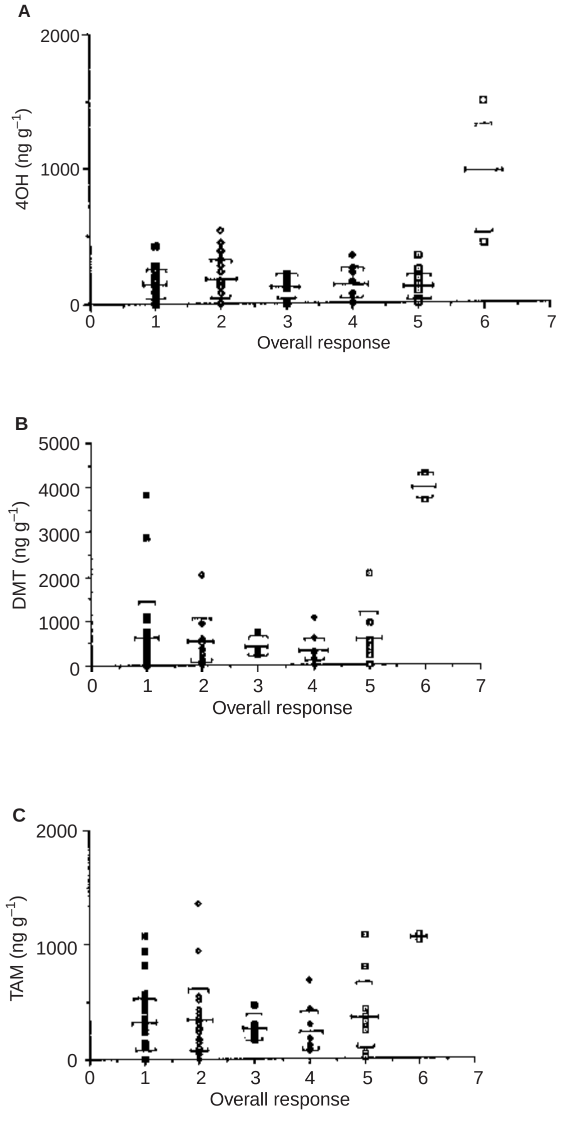

**Figure 3** Overall response to tamoxifen therapy plotted against drug levels. (**A**) = 4OH; (**B**) = DMT; (**C**) = TAM. Columns relate to tissue levels (mean ± s.d.): 1 = response; 2 = acquired resistance; 3 = de novo resistance; 4 = mucoid pathology; 5 = ER-poor status; 6 = Lymph nodes

**Figure 4** Inter-relationships between metabolites. (**A**) TAM vs 4OH in de novo resistant tumours ($r$ = 0.591); (**B**) TAM vs DMT in responding tumours ($r$ = 0.598); (**C**) TAM vs DMT in acquired resistance tumours ($r$ = 0.802)

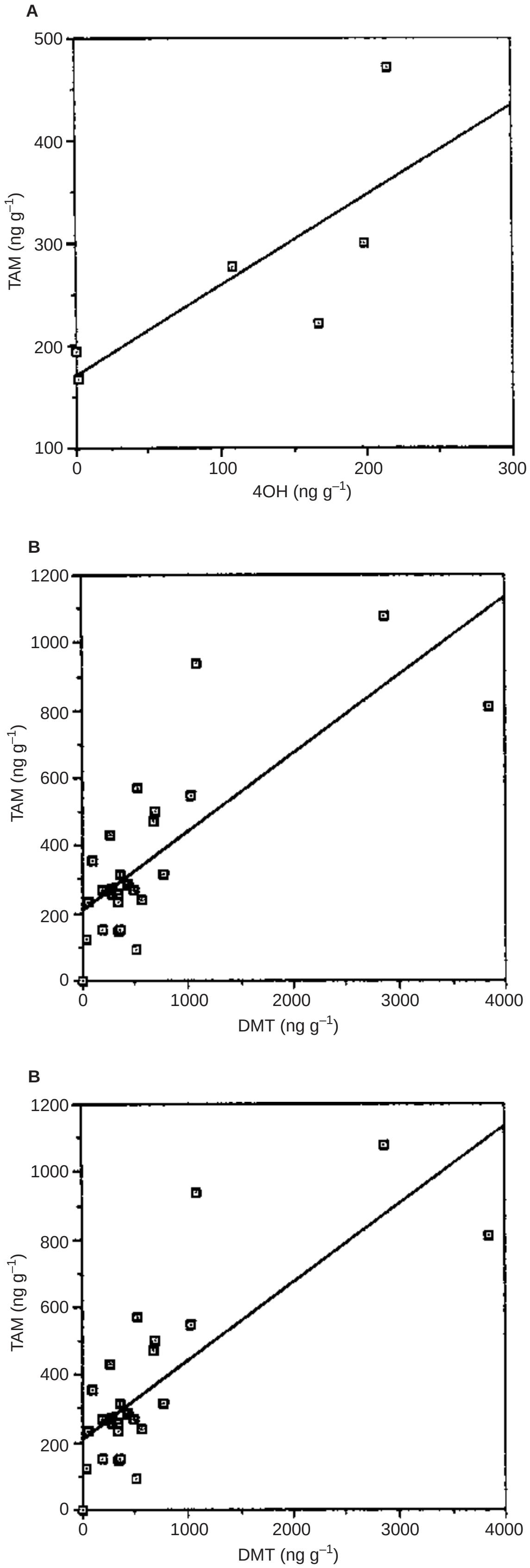

**Figure 4** Inter-relationships between metabolites. (**A**) TAM vs 4OH in de novo resistant tumours ($r$ = 0.591); (**B**) TAM vs DMT in responding tumours ($r$ = 0.598); (**C**) TAM vs DMT in acquired resistance tumours ($r$ = 0.802)

 *© 2000 Cancer Research Campaign*

**Table 3** Mean levels of TAM and metabolites in plasmas and tumour tissues from patients correlated with overall response to therapy

| Tumour[a] | RR (*n* = 25) | RNR (*n* = 22) | NRNR (*n* = 6) | LN (*n* = 2) | Mucoid (*n* = 8) | ER (*n* = 8) |
|---|---|---|---|---|---|---|
| 4OH | 138 (± 107) | 179 (± 165) | 115 (± 96) | 960 (± 739) | 131 (± 134) | 115 (± 122) |
| DMT | 610 (± 875) | 566 (± 549) | 427 (± 234) | 3989 (± 438) | 346 (± 335) | 586 (± 604) |
| TAM | 335 (± 233) | 345 (± 303) | 272 (± 110) | 1054 (± 37) | 249 (± 215) | 399 (± 340) |
| Plasma[b] | | | | | | |
| 4OH | 68 (± 30) | 64 (± 40) | 74 (± 23) | 59 (± 53) | 69 (± 34) | 33 (± 26) |
| DMT | 224 (± 98) | 230 (± 135) | 313 (± 80) | 562 (± 620) | 194 (± 121) | 136 (± 61) |
| TAM | 118 (± 58) | 116 (± 70) | 131 (± 30) | 207 (± 239) | 106 (± 87) | 68 (± 39) |

[a]ng $g^{-1}$ ± s.d. [b]ng $ml^{-1}$ ± s.d.

(Mann–Whitney, $P$ = 0.01–0.03). Similarily, levels of DMT in ER-poor patient plasmas were significantly lower than in both responding and de novo resistant patients (Mann–Whitney, $P$ = 0.03, $P$ = 0.0003), whilst TAM levels in this group were significantly less than those in the de novo resistant group (Mann–Whitney, $P$ = 0.01).

### DISCUSSION

The aim of this study was to measure levels of TAM, 4OH and DMT in plasma and tumour tissue taken from patients undergoing primary neoadjuvant therapy with TAM, and to correlate these with the response of individual tumours to treatment. After an initial tumour biopsy, patients were treated with TAM for at least 3 months, during which time the response of the remaining tumour was monitored. Thus, we have been able to subdivide patients not only as to whether their tumours exhibited resistance to TAM or not, but also whether this was de novo, or acquired resistance. We hoped to discover if differences in levels of TAM and its major metabolites could account for differences in tumour response.

Levels of TAM and metabolites in both plasma and tumour tissue compared well with those previously reported, although there was a tendency for levels of 4OH and DMT to be somewhat higher than those detected by other investigators (Daniel et al, 1979; Robinson et al, 1989; Langan-Fahey et al, 1990; Lim et al, 1993; MacCallum et al, 1997). There could be several explanations for this: from recovery of spiked plasmas, 4OH as the first metabolite off the HPLC column was seen to be the metabolite most likely to be contaminated with spurious peaks, thus overestimation of plasma levels of all three metabolites, most especially 4OH (as the metabolite present at the lowest concentrations) is a possibility. Alternatively, high levels of this metabolite could reflect differences in patient population (taking into account those patients with responding as well as non-responding tumours); with many patients undergoing long-term TAM therapy, levels could reflect accumulation of 4OH with time, for which there is some evidence with regard to tumour tissue (Table 1 and Results), particularly in RNR patients (Figure 2).

On statistical analysis, where tumour levels of TAM, 4OH and DMT were correlated with response to therapy, whether at 3 months, at the time of final surgery or overall, there were no significant differences which would account for the development of resistance to TAM, although differences in the mean and median levels of each metabolite did exist. However, the range of values in each group was wide (see Tables 1–3), thereby nullifying the statistical strength of these differences. Whilst this is a likely reflection of the reproducibility of our assay (as constrained by tissue availability), it may also reflect the heterogeneity of breast tumours, and could relate to tumour cellularity. Whilst we attempted to minimize the effects of heterogeneity, we were unable to correct for cellularity within our sample group. It is interesting to note that high levels of metabolites were found in two lymph node metastases sampled (Table 3), as these are highly cellular.

Investigations on inter-relationships between metabolites carried out by plotting the tumour concentration of one against the others show some interesting data (Figure 4.). Concentrations of TAM and 4OH were significantly associated in de novo resistant tumours, suggesting that levels of TAM in these tumours could indicate similar trends in levels of 4OH, whilst responding and acquired resistance tumours showed significant associations between TAM and DMT levels. These results could indicate fundamental differences in the metabolism of TAM within the different response groups.

On analysis of plasma levels, some significant differences were noted, particularly after 3 months of TAM treatment. At this time, plasma levels of both 4OH and DMT in responding patients were found to be significantly lower than those in non-responding patients (Table 2), whilst plasma DMT levels were significantly higher in de novo resistant patients when compared to responding patients (Table 3). These differences could indicate increased metabolism of TAM in non-responding patients, or increased uptake of these metabolites into tumour tissues in responding patients. Certainly, there is evidence for accumulation of 4OH in tumour tissues of the acquired resistance group with increasing treatment time.

Our results are contrary to the decreased accumulation of tamoxifen found in tumours showing acquired resistance, which has been reported by several investigators (Osborne et al, 1987, 1991, 1992; Johnstone et al, 1993). There could be several reasons for this. We mention previously that differences in the population of patients involved in the study, with the inclusion of both responding and non-responding patients, may have had an effect, and we have not involved ER-poor patients in the whole analysis as others have. The inclusion in the present study of a substantial number of patients on long-term TAM therapy may confound, hiding the predominantly short-term effects reported on previously.

On analysis of a small number of ER-poor patients ($n$ = 8, 1–3 months TAM), we found significantly lower plasma levels of each metabolite when compared to certain of the other response groups (Table 3 and Results). This could be indicative of the short TAM treatment given to these patients, although there is no such

© 2000 Cancer Research Campaign 

evidence for such low levels of plasma in ER-rich patients receiving equally brief treatment (Table 1, < 5 months treatment). Indeed, it is thought that steady-state levels of the drug are reached within 1 month of treatment (Langhan-Fahey et al, 1990; Robinson and Jordan, 1994). Although ER-poor tumour levels were seen to be compatable with the other groups, low levels in plasma may indicate differences in uptake or metabolism of TAM in these patients from the commencement of treatment. Certain markers of tumour growth can be examined and proven predictive of response prior to the achievement of steady-state TAM levels (Cameron et al, 1997). Early differences in levels of, or accumulation of, TAM and metabolites may yet prove to be of importance.

Where tumour levels were evaluated against overall response, no significant differences in uptake were noted which would account for differences in response. However, where length of treatment was also considered, tumour tissue 4OH levels accumulated significantly over time (Table 1 and Results), especially in the subset of tumours showing acquired resistance (Figure 2). As these patients had undergone prolonged TAM therapy, this could indicate a slow change within the tumour, such as clonal expansion of resistant cells, or a change in tumour cell adaptive processes thereby causing a switch from response to resistance in the presence of a highly potent anti-oestrogen (100–1000 times stronger than TAM).

In a previous study involving an overlapping group of patients treated at the Edinburgh Breast Unit, there was evidence of a correlation between TAM levels and weight (Love et al, 1997), although no previous correlations between TAM or DMT levels and obesity have been found (Langhan-Fahey et al, 1990). Analysis of the data for patients whose weight at presentation was known, and for whom a ratio of tumour or plasma concentration $kg^{-1}$ was produced, showed no significant differences between response groups.

In this study we did not set out to obtain evidence for tamoxifen-stimulated tumour growth, such as measuring the amount of metabolite E (Osborne, 1995), as we were looking for differences in metabolism or drug accumulation which could account for resistance to therapy. From our study, it would appear that knowledge of tumour levels of TAM, 4OH and DMT does not correlate with response to TAM therapy. In certain subgroups of our study group, both plasma and tumour levels may indeed have relevance to response, if taken in combination with other factors such as length of treatment.

### REFERENCES

Campbell DA, Ritchie AA, Langdon S, Anderson TJ and Miller WR (1997) Tamoxifen induced apoptosis in R-75 breast cancer xenografts antedates tumour regression. *Breast Cancer Res Treat* **45**: 99–107

Daniel CP, Gaskell SJ, Bishop H and Nicholson RI (1979) Determination of tamoxifen and an unhydroxylated metabolite in plasma from patients with advanced breast cancer using gas chromatography mass spectometry. *J Endocrinol* **83**: 401–408

Daniel P, Gaskell SJ, Bishop H, Campbell C and Robertson RI (1981) Determination of tamoxifen and biologically active metabolites in human breast tumours and plasma. *Eur J Cancer Clin Oncol* **17**: 1183–1189

Forouhi P, Walsh JS, Anderson TJ and Chetty U (1994) Ultrasonography as a method of measuring breast tumour size and monitoring response to primary systemic therapy. *Br J Surg* **81**: 223–225

Foster BD, Cavener DR and Parl FF (1991) Binding analysis of the oestrogen receptor to its specific DNA target site in human breast cancer tissue. *Cancer Res* **51**: 3405–3410

Gottardis MM and Jordan VC (1988) Development of tamoxifen-stimulated growth of MCF-7 tumours in athymic mice after long-term antioestrogen administration. *Cancer Res* **48**: 5183–5187

Gottardis MM, Jiang SY, Jeng MH and Jordan VC (1989) Inhibition of tamoxifen stimulated growth of an MCF-7 variant in athymic mice by novel steroidal antioestrogens. *Cancer Res* **49**: 4090–4093

Graham ML, Krett NL, Miller LA, Leslie KK, Gordon DF, Wood WM and Wei LL (1990) T47DCo cell, genetically unstable and containing oestrogen receptor mutations, are a model for the progression of breast cancer to hormone resistance. *Cancer Res* **50**: 6208–6217

Horwitz KB (1993) Can hormone ‘resistant’ breast cancer cells be inappropriately stimulated by tamoxifen? *Ann NY Acad Sci* **684**: 63–74

Johnstone SRD, Haynes BP, Smith IE, Jarman M, Sacks NPM, Ebbs SR and Dowsett M (1993) Acquired tamoxifen resistance in human breast cancer and reduced intra-tumoral drug concentration. *Lancet* **342**: 1521–1522

Katzenellenbogen BS, Miller MA, Mullick A and Sheen YT (1985) Antioestrogen action in breast cancer cells: modulation of proliferation and protein synthesis, and interaction with oestrogen receptors and additional antioestrogen binding sites. *Breast Cancer Res Treat* **5**: 231–243

Koga M and Sutherland RL (1987) Epidermal growth factor partially reverses the inhibition effects of antioestrogens on T47D human breast cancer cell growth. *Biochem Biophys Res Commun* **146**: 739–745

Langan-Fahey SM, Tormey DC and Jordan VC (1990) Tamoxifen metabolites in patients on long-term adjuvant therapy for breast cancer. *Eur J Cancer* **26** (8): 883–888

Lerner LJ and Jordan VC (1990) Development of antioestrogens and their use in breast cancer. *Cancer Res* **50**: 4177–4189

Lien EA, Solheim E and Ueland PM (1991*a*) Distribution of tamoxifen and its metabolites in rat and human tissues during steady-state treatment. *Cancer Res* **51**: 4837–4844

Lien EA, Webster K, Lonning PE, Solheim E and Ueland PM (1991*b*) Distribution of tamoxifen and metabolites into brain tissue and brain metastases in breast cancer patients. *Br J Cancer* **63**: 641–645

Lim CK, Chow LCL, Yuan Z-X and Smith LL (1993) High performance liquid chromatography of tamoxifen and metabolites in plasma and tissues. *Biomed Chromatogr* **7**: 311–314

Love CDB, Muir BB, MacCallum J, Miller WR, Scrimgeour JB, Leonard RCS and Dixon JM (1997) Relation between tamoxifen duration and endometrial thickness. *Breast Cancer Res Treat* **46** (Suppl): 26

MacCallum J, Cummings J, Dixon JM and Miller WR (1996) Solid-phase extraction and high-performance liquid chromatographic determination of tamoxifen and its major metabolites in plasma. *J Chromatogr B* **678**: 317–323

MacCallum J, Cummings J, Dixon JM and Miller WR (1997) Solid-phase extraction and high-performance liquid chromatographic determination of tamoxifen and its major metabolites in breast tumour tissues. *J Chromatogr B* **698**: 269–275

Miller MA and Katzenellenbogen BS (1983) Characterization and quantitation of antioestrogen binding sites in oestrogen receptor-positive and negative human breast cancer cell lines. *Cancer Res* **43**: 3094–3100

Miller MA, Lippman ME and Katzenellenbogen BS (1984) Antioestrogen binding in antioestrogen growth resistant oestrogen-responsive clonal variants of MCF-7 human breast cancer cells. *Cancer Res* **44**: 5038–5045

Murphy LC and Dozlaw H (1989) Variant oestrogen receptor mRNA species detected in human breast cancer biopsy samples. *Mol Endocrinol* **3**: 687–693

Murphy LC and Sutherland RL (1981) A high affinity binding site for the antioestrogens, tamoxifen and CI 628 in immature rat uterine cytosol which is distinct from oestrogen receptors. *J Endocrinol* **91**: 155–161

Osborne CK (1995) Tamoxifen metabolism as a mechanism for resistance. *Endocrine-related Cancer* **2**: 53–58

Osborne CK, Coronado EB and Robinson JP (1987) Human breast cancer in the athymic nude mouse: cytostatic effect of long-term antioestrogen therapy. *Eur J Cancer Clin Oncol* **23**: 1189–1196

Osborne CK, Coronado E, Allred DC, Wiebe VY and Degregorio M (1991) Acquired tamoxifen resistance: correlation with reduced breast tumour levels of tamoxifen and isomerization of *trans*-4-hydroxytamoxifen. *J Natl Cancer Inst* **83**: 1477–1482

Osborne CK, Wiebe VY, McGuire WL, Ciocca DR and Degregorio MW (1992) Tamoxifen and the isomers of 4-hydroxytamoxifen in tamoxifen-resistant tumours from breast cancer patients. *J Clin Oncol* **10**: 304–310

Raam S, Robert N, Pappas CA and Tamura H (1988) Defective oestrogen receptors in human mammary cancers: their significance in defining hormone dependence. *J Natl Cancer Inst* **80**: 756–761

© 2000 Cancer Research Campaign

Robinson SP and Jordan VC (1988) Metabolism of steroid modifying anticancer agents. *Pharmacol Ther* **36**: 41–103

Robinson SP and Jordan VC (1994) Metabolism of antihormonal anticancer agents. In: *Anticancer Drugs: Antimetabolite Metabolism and Natural Anticancer Agents*, Powlis C (ed), pp. 279–289 (section 5.2). Pergamon Press: Oxford

Robinson SP, Langan-Fahey and Jordan VC (1989) Tamoxifen metabolism in the athymic mouse: implications for the study of antitumour effects upon human breast cancer xenografts. *Eur J Cancer Clin Oncol* **25**: 1769–1776

Scott GK, Kushner P, Vigne P and Benz CC (1991) Truncated forms of DNA-binding oestrogen receptor. *J Clin Invest* **88**: 700–706

Tormey DC, Simon R, Lippman ME, Bull JM and Myers CE (1976) Evaluation of tamoxifen dose in advanced breast cancer: a progress report. *Cancer Treat Rep* **60**: 1451–1459

Wolf DM, Langan-Fahey SM, Parker CJ, McCague R and Jordan VC (1993) Investigation of the mechanism of tamoxifen-stimulated breast tumour growth with non-isomerizable analogues of tamoxifen and metabolites. *J Natl Cancer Inst* **85**: 806–812

*© 2000 Cancer Research Campaign*
